# Time-evolving dynamics in brain networks forecast responses to health messaging

**DOI:** 10.1162/netn_a_00058

**Published:** 2018-11-01

**Authors:** Nicole Cooper, Javier O. Garcia, Steven H. Tompson, Matthew B. O’Donnell, Emily B. Falk, Jean M. Vettel

**Affiliations:** Annenberg School for Communication, University of Pennsylvania, Philadelphia, PA, USA; U.S. Army Research Laboratory, Aberdeen Proving Ground, Aberdeen, MD, USA; U.S. Army Research Laboratory, Aberdeen Proving Ground, Aberdeen, MD, USA; Department of Bioengineering, University of Pennsylvania, Philadelphia, PA, USA; U.S. Army Research Laboratory, Aberdeen Proving Ground, Aberdeen, MD, USA; Department of Bioengineering, University of Pennsylvania, Philadelphia, PA, USA; Annenberg School for Communication, University of Pennsylvania, Philadelphia, PA, USA; Annenberg School for Communication, University of Pennsylvania, Philadelphia, PA, USA; U.S. Army Research Laboratory, Aberdeen Proving Ground, Aberdeen, MD, USA; Department of Bioengineering, University of Pennsylvania, Philadelphia, PA, USA; Department of Psychological and Brain Sciences, University of California, Santa Barbara, CA, USA

**Keywords:** Functional MRI (fMRI), Neuroimaging, Functional connectivity, Behavior change, Smoking

## Abstract

Neuroimaging measures have been used to forecast complex behaviors, including how individuals change decisions about their health in response to persuasive communications, but have rarely incorporated metrics of brain network dynamics. How do functional dynamics within and between brain networks relate to the processes of persuasion and behavior change? To address this question, we scanned 45 adult smokers by using functional magnetic resonance imaging while they viewed anti-smoking images. Participants reported their smoking behavior and intentions to quit smoking before the scan and 1 month later. We focused on regions within four atlas-defined networks and examined whether they formed consistent network communities during this task (measured as allegiance). Smokers who showed reduced allegiance among regions within the default mode and fronto-parietal networks also demonstrated larger increases in their intentions to quit smoking 1 month later. We further examined dynamics of the ventromedial prefrontal cortex (vmPFC), as activation in this region has been frequently related to behavior change. The degree to which vmPFC changed its community assignment over time (measured as flexibility) was positively associated with smoking reduction. These data highlight the value in considering brain network dynamics for understanding message effectiveness and social processes more broadly.

## Author Summary

How do functional dynamics within and between brain networks relate to the processes of persuasion and behavior change? In this report, we assess brain network dynamics by using fMRI while smokers view antismoking messages, and relate these metrics to smoking behavior and intentions to quit smoking 1 month following the scan. Smokers who showed reduced allegiance (less consistent network communities) among regions within the default mode and fronto-parietal networks also demonstrated larger increases in their intentions to quit smoking. Furthermore, the degree to which the ventromedial prefrontal cortex flexibly changed its community assignment over time was positively associated with later smoking reduction. These data show that metrics of functional network dynamics can provide new information about individual differences in responsiveness to anti-smoking messaging.

## INTRODUCTION

Neural measures have forecasted future changes in behavior across a number of domains (Berkman & Falk, 2013; Gabrieli, Ghosh, & Whitfield-Gabrieli, 2015). This has included clinical treatment outcomes and health (Costafreda, Khanna, Mourao-Miranda, & Fu, 2009; Doehrmann et al., 2013; Feldstein Ewing et al., 2017; Lopez et al., 2017; Wilcox et al., 2017; Yang et al., 2016) as well as changes in individuals’ health behaviors in response to persuasive messaging. Neural activity during health messaging has been associated with reductions in smoking (Chua et al., 2011; Cooper, Tompson, O’Donnell, & Falk, 2015; Falk, Berkman, Whalen, & Lieberman, 2011; Falk et al., 2015; Riddle, Newman-Norlund, Baer, & Thrasher, 2016; Zelle, Gates, Fiez, Sayette, & Wilson, 2017), decreases in sedentary behavior (Cooper, Bassett, & Falk, 2017; Falk et al., 2015), and increased sunscreen use (Falk, Berkman, Mann, Harrison, & Lieberman, 2010; Vezich, Katzman, Ames, Falk, & Lieberman, 2017). These studies have largely related future health behaviors to neural activity in a small number of brain regions. However, these individual regions are also actively communicating with one another by forming dynamic networks to integrate activity across disparate brain regions (Bressler & Menon, 2010; Sporns, Chialvo, Kaiser, & Hilgetag, 2004; Sporns, Tononi, & Edelman, 2000). Consequently, a host of recent research has developed new approaches to studying global patterns in large-scale brain networks and has demonstrated that analyses of networks can provide new insight into brain function and behavior (Bullmore & Sporns, 2009; Friston, 2009; Medaglia, Lynall, & Bassett, 2015; Menon, 2011).

We examined dynamic functional connectivity among network communities while a group of smokers were exposed to anti-smoking health messaging, and we hypothesized that individual differences in network interactions during messaging would precede subsequent changes in intentions to quit smoking and actual smoking behavior. We focused on four a priori networks which were defined based on resting-state data (Power et al., 2011). Large-scale brain networks can be identified through the analysis of correlated neural activity during rest or during relevant cognitive tasks (Bressler & Menon, 2010; Friston, 1994; Raichle et al., 2001). Regional interactions when the brain is at rest capture its intrinsic architecture (Fox & Raichle, 2007; Greicius, Krasnow, Reiss, & Menon, 2003), and as such, the resulting network communities are thought to impose strong constraints on information processing in the brain (Fox et al., 2005; Power et al., 2011; Shirer, Ryali, Rykhlevskaia, Menon, & Greicius, 2012). Network communities identified at rest are relevant for behavior and performance and can be mapped on to broad categories of cognitive processes (Smith et al., 2009); for example, dynamic changes in interactions among these network communities can account for performance variability (Bassett, Wymbs, et al., 2013; Bassett, Yang, Wymbs, & Grafton, 2015; Braun et al., 2015; Deng, Chandrasekaran, Wang, & Wong, 2016; Gerraty et al., 2018; Liang, Zou, He, & Yang, 2016; Wang, Ong, Patanaik, Zhou, & Chee, 2016). Thus, we argue that networks defined during the resting state identify fundamentally related systems of regions, which are functionally relevant and predictive of task performance. We examine dynamics in these networks during task performance to demonstrate a link between individual differences in health message processing and later smoking-related outcomes.

More specifically, we focused on four a priori networks of interest whose regions have been associated with processes relevant for behavior change in previous research: the default mode, fronto-parietal control, salience, and subcortical networks (Falk & Scholz, 2017; Kaye, White, & Lewis, 2017). The default mode network is thought to form a system for self-related cognitive processing, including social processing, memory, and prospection (Bressler & Menon, 2010; Buckner et al., 2009; Laird et al., 2011). The salience network is critical for selecting and responding to behaviorally relevant stimuli (Barrett & Satpute, 2013; Menon, 2011; Seeley et al., 2007). A growing body of previous work relating health-related outcomes to brain activity has implicated individual brain regions that are part of the default mode and salience networks (Chua et al., 2011; Cooper et al., 2017; Dinh-Williams, Mendrek, Dumais, Bourque, & Potvin, 2014; Falk et al., 2015; Ramsay, Yzer, Luciana, Vohs, & MacDonald, 2013; Riddle et al., 2016; Vezich et al., 2017; Wang et al., 2013; Weber, Huskey, Mangus, Westcott-Baker, & Turner, 2015; Zelle et al., 2017); future behavior has also been related to task activation in the striatum (Berns & Moore, 2012; Genevsky & Knutson, 2015; Kühn, Strelow, & Gallinat, 2016; Venkatraman et al., 2015). Finally, changes in the fronto-parietal control network, thought to support task-switching, have been linked to learning and decision-making (Bassett et al., 2011; Braun et al., 2015; Gerraty et al., 2018), processes which are likely to be relevant to belief updating when receiving new information. Based on the critical role of these cognitive systems in support of behavior change, we hypothesized that better understanding the interactions among the regions in these a priori, atlas-defined networks would uncover an important and yet unstudied component of brain dynamics that can forecast critical health outcomes, changes in intentions to perform a behavior and actual changes in that behavior. We note that individual differences in network dynamics during the task could be due to a trait-like intrinsic difference in network dynamics, or context-dependent differences in how individual smokers process the experience of a smoker viewing anti-smoking messages (which may stem in part from properties of the messages themselves); effects observed here could be due to one or a combination of these possibilities.

Previous research has found that although mean activation in ventromedial prefrontal cortex (vmPFC) is associated with subsequent behavior change, this same brain activity is often uncorrelated with participants’ self-reported intentions (Cooper et al., 2015; Falk et al., 2010, 2011). Several theories of health behavior posit that intentions to perform a behavior are an important precursor to behavior change, but that other factors also influence whether behavior change occurs (Ajzen, 1985, 1991; Armitage & Conner, 2001; Fishbein, 1979; Fishbein & Ajzen, 2011; Webb & Sheeran, 2006). In short, although related, intentions to change and actually changing behavior may be associated with partially differing neural precursors. To further explore this possibility, we compare both outcomes (changes in intentions and behavior) to network dynamics.

Although previous research has identified the regions in the default mode, fronto-parietal control, salience, and subcortical networks as key components of successful behavior change, little work has examined how they work in concert. To assess the variable interactions between brain regions in these networks, we utilized two complementary metrics recently developed in network science to quantify regional dynamics, allegiance and flexibility. We first tested whether sustained coordinated processing within regions in the default mode, fronto-parietal, salience, and subcortical networks results in lasting changes in message-consistent outcomes. The extent to which regions form a cohesive community and demonstrate the same pattern of activity across time can be quantified by *allegiance*, where higher allegiance in a network would indicate more sustained coordination of activity and processing within nodes in that network and decreased allegiance would indicate greater diversity in processing across nodes. We thus compare individuals’ changes in smoking-relevant outcomes to the allegiance in four key brain networks during messaging.

We further examined the vmPFC specifically, which is the region most consistently associated with future behavior change in previous work (Chua et al., 2011; Cooper et al., 2015; Falk et al., 2010, 2015; Falk & Scholz, 2017; Riddle et al., 2016; Vezich et al., 2017). Given the vmPFC’s role in integrating multiple sources of information to compute a value signal (Bartra, McGuire, & Kable, 2013), we propose that successful change in behavior requires dynamic connections between the vmPFC and other relevant cognitive systems, which will be indexed by increased vmPFC *flexibility*. This measure focuses on the activity of single brain regions, revealing whether a region remains a member of the same community over time or if it frequently (and flexibly) changes its assignment across communities between time points. Thus, we test the importance of both consistent interactions among regions and dynamic changes between networks during messaging about smoking cessation.

## METHODS

### Participants

The study sample consisted of 45 participants (28 men; mean age = 32 years, *SD* = 13; 30 White). All participants gave written, informed consent in accordance with the procedures of the Institutional Review Board at the University of Michigan. Of the original 50 participants, 2 participants were excluded for missing data (1 due to an error at the scanner, and another for not participating in the final session). Three participants were excluded for data quality issues (1 for neurological abnormalities, 1 for excessive head motion, and a third for both vision problems and excessive head motion).

Participants were recruited from the general population by using Craigslist and a university website. Initial eligibility was assessed through a phone call. To be eligible, potential participants must have been between the ages of 18 and 65, have smoked at least 5 cigarettes per day for the past month, and have been a smoker for at least 12 months. In addition, participants had to meet standard fMRI eligibility criteria, including having no metal in their body, no history of psychiatric or neurological disorders, and currently not taking any psychiatric or illicit drugs.

### Study Timeline and Measures

Following a screening for eligibility via telephone, participants completed three study sessions. The first session (Session 1) provided baseline measures of self-reported smoking behavior and intentions to quit or reduce smoking, which were reported again at each following session. The fMRI scan (Session 2) took place an average of 6 days later. The follow-up session (Session 3) was conducted via telephone, an average of 39 days after Session 2.

### Smoking Outcomes

We assessed two smoking outcomes and their relation to neural dynamics. We first examined changes in intentions to quit smoking. At each of the three sessions, participants were asked three questions about their intentions to quit, reduce, or refrain from smoking in the next three months. The intention ratings were made on a four-point scale (anchors: 1 = definitely will not, 2 = probably will not, 3 = probably will, and 4 = definitely will). Responses to these questions were averaged for each time point. Intention change for each individual was measured as the difference between the average of all intention questions at Session 1 and the average at Session 3. Intentions were also measured immediately after the scan (Session 2), but intention change from Session 1 to Session 2 was not associated with network measures or behavior change.

We also examined changes in self-reported smoking behavior. Participants were asked to report the number of cigarettes they smoked per day at each of the three study sessions. As a reference, they were told that a pack contains 20 cigarettes. We related neural dynamics to the percent change in cigarettes smoked per day from Session 1 to Session 3 in each individual. We started with self-reports at Session 1 to match the time point of the intention measure; the reports of daily smoking at Session 1 and Session 2 were very consistent (*r* = 0.94). Self-report measures are commonly used to track smoking behavior change (Chua et al., 2011; Jasinska et al., 2012) and have been shown to have a moderate to high correlation with physiological metrics such as expired CO (Falk et al., 2011; Jarvis, Tunstall-Pedoe, Feyerabend, Vesey, & Saloojee, 1987; Middleton & Morice, 2000) and saliva and serum cotinine (Etter, Vu Duc, & Perneger, 2000; Patrick et al., 1994; Pokorski, Chen, & Bertholf, 1994; Vartiainen, Seppälä, Lillsunde, & Puska, 2002).

### fMRI Task

**Figure 1.**
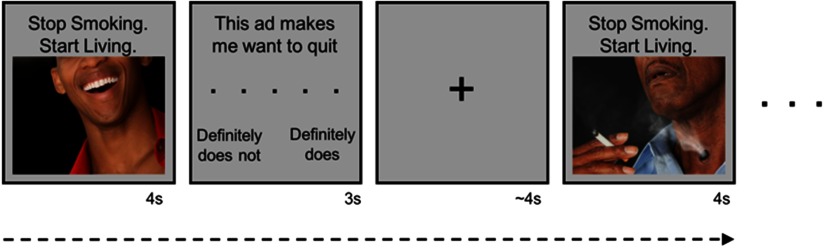
Task design. While undergoing fMRI, participants viewed images paired with the tagline “Stop Smoking. Start Living.”

Participants completed four tasks in the scanner, but this analysis focused on the main task of interest, a persuasive messaging task that promoted smoking cessation. Participants saw 80 images with the tagline “Stop Smoking. Start Living.” Each trial consisted of 4 s of image presentation, followed by a 3-s response screen with the statement “This makes me want to quit” and a five-point rating scale (1 = definitely does not, 5 = definitely does); see Figure 1. The response period was followed by a jittered intertrial interval, consisting of a screen with only a fixation cross (3–7.5 s, mean = 4.10 s, median = 3.32 s, *SD* = 1.01 s).

Participants viewed 30 negative anti-smoking images, based on the FDA’s proposed graphic warning labels. Of these, 12 portrayed social consequences of smoking (e.g., exclusion from a group) and 18 portrayed nonsocial and health-related consequences of smoking (e.g., a tracheotomy). Additionally, participants viewed 30 neutral control images (11 social, 19 nonsocial). The negative and neutral images were qualitatively matched in pairs, by overall composition of the content (e.g., X-ray image of a diseased lung and X-ray image of a healthy lung), focal point, and number of people in the image. The remaining 20 face images were a between-subject manipulation of personalization, where one set of participants saw images of their Facebook friends (*N* = 19 participants) and the other (*N* = 26 participants) saw unknown faces from a public database known as NimStim (Tottenham et al., 2009). We controlled for this between-subject manipulation in the regression analyses below, confirming that it was not significantly related to outcomes of interest. Each image was presented once, and the order of image presentation was randomized across individuals.

### MRI Data Acquisition

Neuroimaging data were acquired using a 3 Tesla GE Signa MRI scanner. Two functional runs of one task (454 volumes total) are analyzed here. Functional images were recorded using a reverse spiral sequence (TR = 2,000 ms, TE = 30 ms, flip angle = 90°, 43 axial slices, FOV = 220 mm, slice thickness = 3 mm; voxel size = 3.44 × 3.44 × 3.0 mm). We also acquired in-plane T1-weighted images (43 slices; slice thickness = 3 mm; voxel size = .86 × .86 × 3.0 mm) and high-resolution T1-weighted images (SPGR; 124 slices; slice thickness = 1.02 × 1.02 × 1.2 mm) for use in coregistration and normalization.

### fMRI Preprocessing

Functional data were preprocessed and analyzed using Statistical Parametric Mapping (SPM8, Wellcome Department of Cognitive Neurology, Institute of Neurology, London, UK). To allow for the stabilization of the BOLD signal, the first five volumes (10 s) of each run were not recorded by the scanner. Functional images were despiked using the 3dDespike program (AFNI; Cox, 1996). Next, data were corrected for differences in the time of slice acquisition by using sinc interpolation, where the first slice served as the reference slice. Data were then spatially realigned to the first functional image. We then coregistered the functional and structural images by using a two-stage procedure. First, in-plane T1 images were registered to the mean functional image. Next, high-resolution T1 images were registered to the in-plane T1 image. After coregistration, high-resolution structural images were segmented to produce a gray matter mask, and then normalized to the skull-stripped MNI template provided by FSL. Finally, functional images were smoothed using a Gaussian kernel (8-mm full width at half maximum).

Based on preliminary recent evidence suggesting the possible sensitivity of network results to spatial smoothing (Alakörkkö, Saarimäki, Glerean, Saramäki, & Korhonen, 2017; Chen & Calhoun, 2018), we conducted comparative analyses with unsmoothed data and confirmed that both the regional time course dynamics and a region’s temporally evolving community affiliation were highly similar across smoothed and unsmoothed data in this study. We repeated the main analyses below with unsmoothed data, and present these results in the Supporting Information (Cooper, Garcia, Tompson, O’Donnell, Falk, & Vettel, 2018).

### Functional Connectivity Analysis

**Figure 2.**
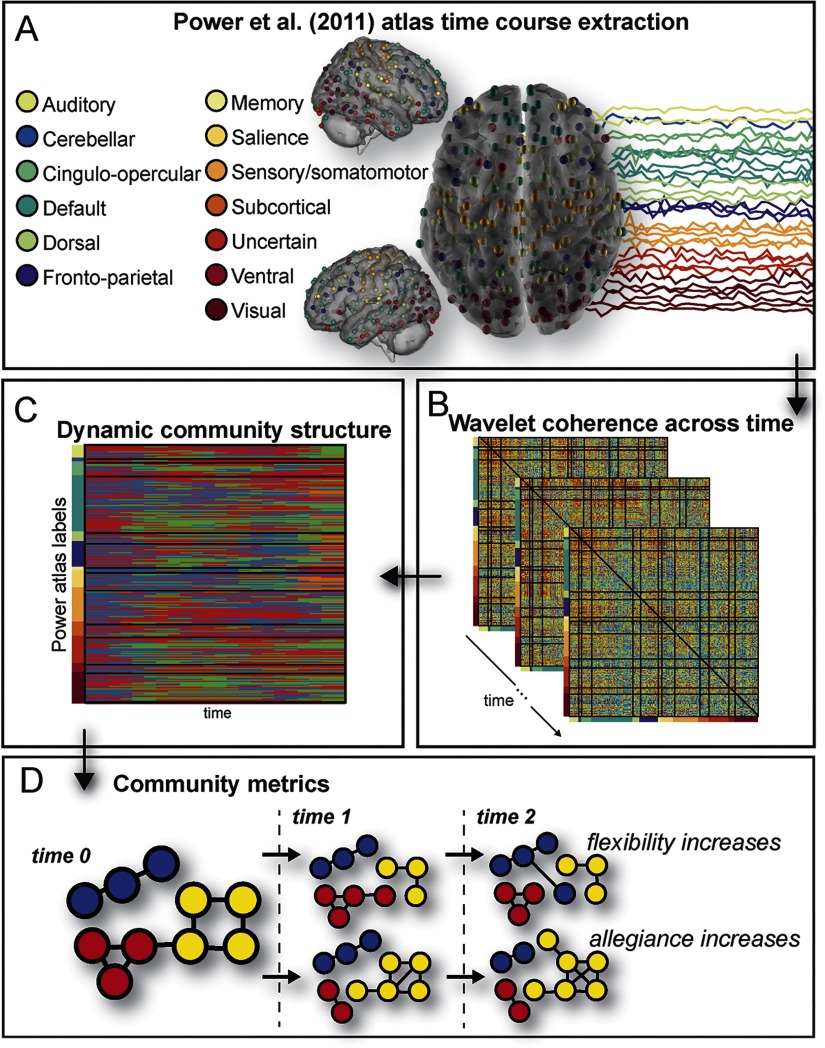
Analysis design: overview of analysis scheme. We extracted the time series of activation in all nodes of the Power atlas brain parcellation during the task (A). Using wavelet coherence as a measure of functional connectivity (B) and input to a dynamic community detection algorithm (C), we explored community affiliations across the time course of the task using two metrics, flexibility and allegiance, which are explained in a hypothetical network (D). From an initial network configuration at *time 0*, regions reconfigure over time. In the top row, a node changes its affiliation from the yellow community at *time 0* to the red community at *time 1*, then to the blue community at *time 2*, indicating increased flexibility relative to nodes remaining in the same community at all time points. In the bottom row, the yellow community gains more nodes and more connections between nodes across time, indicating increased allegiance.

Following preprocessing, the mean signal was extracted from 264 atlas-defined regions of interest (ROIs) by using the MarsBar package for SPM. These ROIs were spherical regions with an 8-mm radius, centered on the 264 coordinates defined by Power et al. (2011). The detrended time courses from these regions were divided into 22 nonoverlapping bins of 20 TRs (where 20 TRs = 40 s); this bin size was chosen to optimize the detection of individual differences in dynamics during the task (Telesford et al., 2016). Given the short event–related design of this task, and relatively small number of images in each task condition, we did not compare dynamics of connectivity across the task separately by task condition. Wavelet coherence was estimated in each bin for each pair of regions, and was averaged across frequency bands between 0.06 and 0.12 Hz, a task-relevant frequency range of coherence (Sun, Miller, & D’Esposito, 2004). This resulted, for each bin, in a 264 × 264 matrix of coherence values for each pair of regions (Figure 2B). These 264 regions are identified by Power et al. (2011) as composing 13 networks, depicted in Figure 2A. Based on previous research, our analysis focused on four networks from the Power atlas: the default mode, fronto-parietal, salience, and subcortical networks. These networks have been associated with processing indicative of persuasion and successful behavior change (Bassett et al., 2011; Braun et al., 2015; Chua et al., 2011; Cooper et al., 2017; Dinh-Williams et al., 2014; Falk et al., 2015; Gerraty et al., 2018; Ramsay et al., 2013; Riddle et al., 2016; Vezich et al., 2017; Wang et al., 2013; Weber et al., 2015; Zelle et al., 2017).

### Community Detection and Network Metrics

We employed recent advancements from network science to examine whether the synchrony within a network community (allegiance among the brain regions in the same community) or interactions between network communities (flexibility of brain regions to coordinate across communities) accounted for lasting changes in smoking outcomes. To capture changes in network communities over the course of the task, we utilized a multilayer community detection analysis (Bassett et al., 2011; Mucha, Richardson, Macon, Porter, & Onnela, 2010). This allows for the investigation of changes in network structure over time by coupling nodes between adjacent time slices, and results in a community partition for each time window (Figure 2C). The algorithm utilized a generalized Louvain algorithm to optimize modularity (Bassett, Porter, et al., 2013; Telesford et al., 2016). We repeated this optimization 100 times, since the algorithm is nondeterministic and susceptible to near degeneracies (Good, de Montjoye, & Clauset, 2010), and we averaged the iterations to compute the community metrics.

The resulting community structures were used to estimate flexibility and allegiance (Ashourvan, Gu, Mattar, Vettel, & Bassett, 2017). Allegiance is defined as the proportion of time windows during which each pair of nodes were assigned to the same community. Flexibility is defined as the proportion of time windows during which each node changes community assignment. As shown in Figure 2D, the central region shows high flexibility as it changes assignment from the yellow community to the red community at *time 1* and the blue community at *time 2*. In contrast, allegiance identifies regions that are strongly connected over time, as demonstrated by the yellow community in Figure 2D. We employed these two metrics to examine the relationship between brain activity and health outcomes.

### Relating Network Allegiance Metrics and Smoking-Related Outcomes

In our first set of analyses, we examined the relationship between network allegiance measures and changes in smoking-related outcomes. We tested these relationships in the four atlas-defined networks of interest (default mode, fronto-parietal, salience, and subcortical networks). For analyses in the a priori networks, allegiance of all node pairs was averaged to obtain a composite measure of allegiance within the atlas-defined network. In separate models for each a priori network, we used robust regression to predict changes in smoking intentions and percent changes in daily smoking. We examined average allegiance both as a linear metric and binned into quartiles to identify robust trends in the community dynamics (Lange, Oostenveld, & Fries, 2013; van Dijk, Schoffelen, Oostenveld, & Jensen, 2008), where quartile labels were entered as a categorical variable in the robust regression model.

We used the robust regression (RLM) function in R’s (version 3.2.4) MASS library. The Wald test was used to assess significance of RLM coefficients (robtest, R’s sfsmisc package). All models controlled for personalization condition (Facebook vs. NimStim faces), gender, age, and ethnicity (White vs. other); models predicting intention change also controlled for Session 1 (baseline) intentions. Robust linear models are less sensitive to outliers and high leverage data points, allowing the inclusion of all data points. Personalization condition, a between-participants variable, did not significantly relate to the main outcomes discussed in this investigation (metrics of network allegiance and flexibility or smoking-related outcomes).

### Relating Network Flexibility Metrics and Smoking-Related Outcomes

In our final analysis, we examined the relationship between vmPFC flexibility and changes in smoking-related outcomes. We examined vmPFC flexibility both binned into quartiles to test categorical differences (categorical predictor in regression) and as a linear metric. We used robust regression to relate vmPFC flexibility to changes in smoking intentions and percent changes in daily smoking. As above, these models controlled for task condition (Facebook vs. NimStim faces), gender, age, and ethnicity (White vs. other); models predicting intention change also controlled for Session 1 (baseline) intentions.

## RESULTS

In this study, we examine how the dynamics of brain networks during exposure to anti-smoking messaging relate to smoking-related outcomes in the following month. We hypothesized that individual differences in metrics of brain network dynamics during a behaviorally relevant task, rating anti-smoking messages, would precede changes in smoking-related intentions and behavior. Smokers participated in an fMRI scanning session, during which they viewed anti-smoking messages. Before the fMRI scan and 1 month later, participants self-reported their intentions to quit smoking and the number of cigarettes they smoked per day. We first examined brain network dynamics during exposure to anti-smoking messaging in nodes belonging to four a priori networks based on the Power et al. (2011) atlas: the default mode network (DMN), fronto-parietal network (FPN), salience network, and subcortical network. Activation in regions that comprise these networks has been previously linked to persuasion and health behavior change, but their community dynamics have not been investigated. We assessed the functional connectivity between all pairs of regions in 22 consecutive time windows across the course of the task. We then used a dynamic community detection algorithm to study the relationship between brain network dynamics and smoking-related outcomes in two complementary analyses: the first investigated allegiance in our a priori networks of interest, and the second studied flexibility in the vmPFC based on its consistent association with future behavior change in previous work.

### Changes in Smoking Intentions and Behavior

Average intentions to reduce or quit smoking significantly increased from the intake session to the follow-up session (paired *t*(44) = 4.59, *p* < 3.6 × 10^−5^). At Session 1, intentions to quit averaged 2.41 (*SD* = 0.81); at Session 3, intentions to quit averaged 2.97 (*SD* = 0.76). We also examined a second smoking outcome, changes in smoking behavior. At Session 1, participants reported smoking an average of 13.3 (*SD* = 6.5) cigarettes per day. At Session 3, which took place an average of 45 days later, participants smoked an average of 10.2 (*SD* = 7.7) cigarettes per day. This represented a significant decline in the number of cigarettes participants smoked per day from the intake to follow-up session (paired *t*(44) = 3.22, *p* < 0.0024). In the following sections, we examine the relationships between changes in smoking intentions and behavior, and dynamics in neural network measures during exposure to anti-smoking messaging.

### Allegiance in Subnetworks Relates to Changes in Intentions

**Figure 3.**
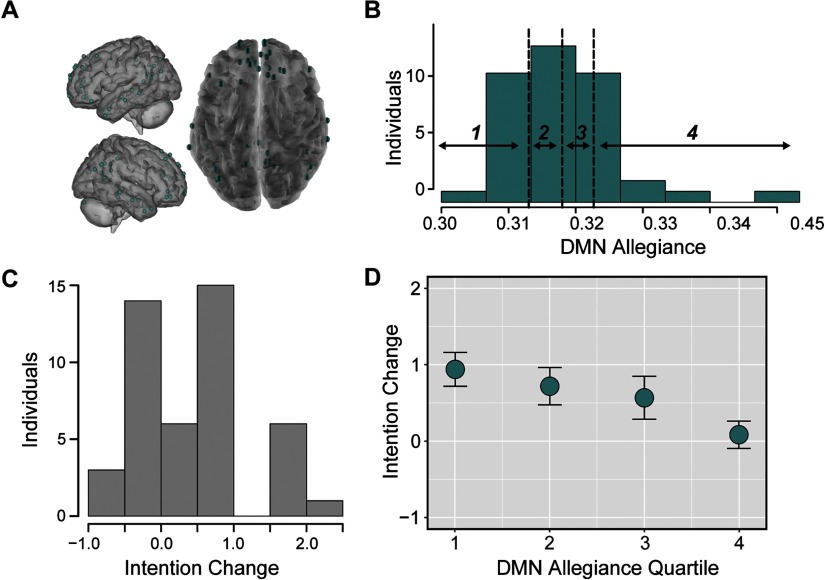
Reduced allegiance within the DMN precedes increased intention change. (A) Nodes in the atlas-defined DMN. (B) Histogram of allegiance between pairs of nodes in the atlas-defined DMN, averaged within individuals. These averages are divided into quartiles, with bin borders noted as vertical dotted lines. (C) Histogram of changes in intentions from Session 1 to Session 3 for each individual, where positive value indicate an increased intention to change over time. (D) Relationship between allegiance of nodes within the atlas-defined DMN and intention change, where intention change was averaged in DMN allegiance quartiles. Error bars represent standard error of the mean.

We first tested whether individual differences in allegiance between nodes within the atlas-defined DMN, a network associated with social processing, self-relevance, valuation, memory, and prospection, were related to message-consistent outcomes after the scanning session. We averaged allegiance between all node pairs in the atlas-defined DMN (Figure 3A) and divided individuals into quartiles based on this distribution (Figure 3B). We then related allegiance in these quartiles to changes in participants’ intentions to quit smoking. A histogram of intention change can be found in Figure 3C. We found that reduced allegiance between nodes within the DMN predicted a greater increase in intentions to quit smoking (quartile robust regression, *t*(38) = −2.86, *p* < 0.007; continuous variable robust regression *t*(38) = −1.99, *p* < 0.049; see Figure 3D and Supporting Information Figure S1, Cooper et al., 2018), controlling for intentions at baseline and demographic covariates. In a parallel analysis, we examined the relationship between allegiance in the atlas-defined DMN and behavior change. In our main analysis, DMN allegiance was not significantly related to reductions in daily smoking (continuous robust regression *t*(39) = 1.41, *p* < 0.167), but these results became significant when examining unsmoothed data (see Supporting Information).

**Figure 4.**
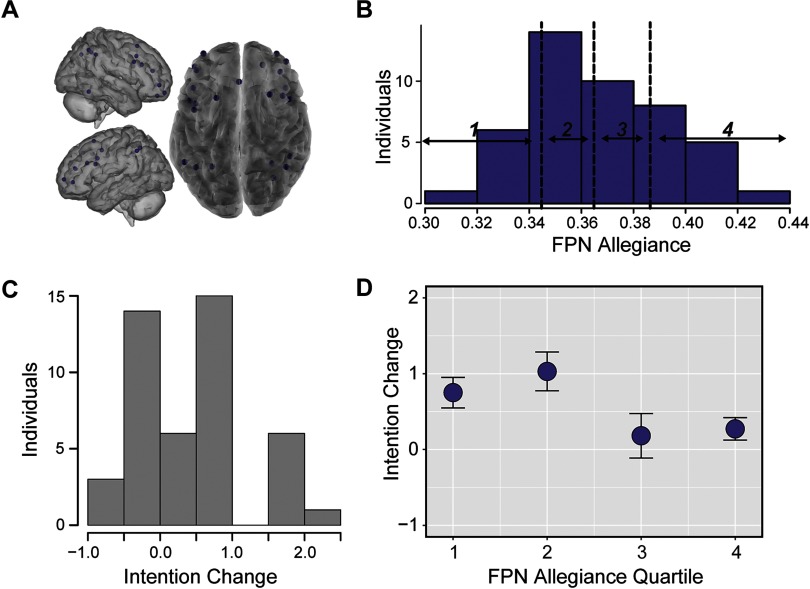
Reduced allegiance within the FPN precedes increased intention change. (A) Nodes in the atlas-defined FPN. (B) Histogram of allegiance between pairs of nodes in the atlas-defined FPN, averaged within individuals. These averages are divided into quartiles, with bin borders noted as dotted vertical lines. (C) Histogram of changes in intentions from Session 1 to Session 3 for each individual. (D) Relationship between allegiance of nodes within the atlas-defined FPN and intention change, where intention change was averaged within FPN allegiance quartiles. Error bars represent standard error of the mean.

We repeated this analysis for the atlas-defined FPN, a network that has been associated with decision-making and may play a critical role in belief updating. Following the same process as DMN, we averaged allegiance between nodes in the FPN (Figure 4A) and divided this distribution into quartiles (Figure 4B). We then related allegiance in these quartiles to changes in participants’ intentions to quit smoking, and our primary results identified that reduced allegiance in the FPN was also related to increased intentions (quartile, *t*(38) = −2.37, *p* < 0.021; continuous variable robust regression *t*(38) = −2.10, *p* < 0.038; see Figure 4D and Supporting Information, Figure S2, Cooper et al., 2018). However, this relationship was trending in the same direction but not significant using unsmoothed data (see Supporting Information). Allegiance in the FPN was not related to reductions in daily smoking (continuous robust regression *t*(39) = 1.19, *p* < 0.238).

We performed parallel analyses in the final two networks of interest, the salience and subcortical networks, for a total of eight tests each of the relationship between network allegiance and intention change, and network allegiance and behavior change for each subnetwork (including the supplemental analyses using unsmoothed data). We found no significant relationships between allegiance and intentions (salience: continuous robust regression, *t*(38) = −0.27, *p* < 0.78; subcortical: continuous robust regression, *t*(38) = −0.11, *p* < 0.91) or reductions in daily smoking (salience: continuous robust regression, *t*(39) = 1.14, *p* < 0.265; subcortical: continuous robust regression, *t*(39) = 1.74, *p* < 0.097).

### vmPFC Flexibility Relates to Later Changes in Behavior

**Figure 5.**
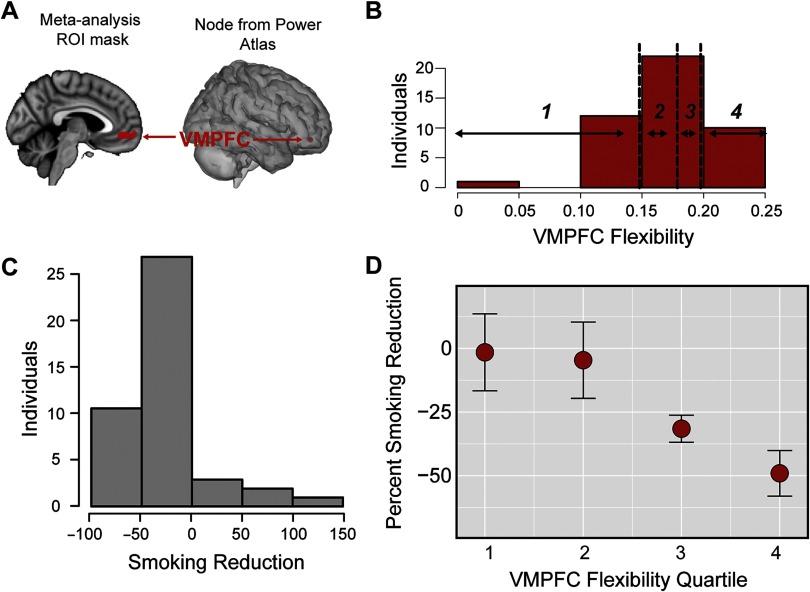
vmPFC flexibility relates to behavior change. (A) Region of vmPFC identified by Falk et al. (2010) (left) and the closest Power parcellation node (right). (B) Histogram of vmPFC flexibility in each individual, with the vertical dotted black line denoting the border for the quartile bins. (C) Histogram of the percent change in cigarettes smoked per day in each individual, where negative values indicate a reduction in cigarettes smoked per day. (D) Relationship between vmPFC flexibility and behavior change, where behavior change was averaged within vmPFC allegiance quartiles. Error bars represent standard error of the mean.

In our final analysis, given its particularly robust presence in the literature on behavior change, we examined whether vmPFC demonstrated coordinated, but flexible, dynamics across multiple network communities. The vmPFC has been posited to be a hub of information processing, integrating inputs about the self-relevance and valuation of information and influencing decision-making, and localized activation in vmPFC has been frequently reported to predict behavior changes following persuasive messaging. To complement these previous activation findings and investigate the possible role of vmPFC in integrating information between multiple network communities, we selected the node in the Power parcellation that was closest to the center of mass of the vmPFC region identified as predictive of behavior change in a sunscreen use study (Falk et al., 2010), shown in Figure 5A; this region has now been used to predict behavior change in several contexts (Cooper et al., 2015; Falk et al., 2011, 2015; Riddle et al., 2016). Of note, the same node is closest to the center of mass of the vmPFC region identified as responding to subjective value in a value-based decision-making meta-analysis by Bartra et al. (2013). This vmPFC node is classified as belonging to the DMN; however, we examined vmPFC separately, as the default mode as defined in the Power atlas is a large network composed of 45 nodes, and thus the behavior of the vmPFC node may not be representative of the entire network (e.g., in past research on behavior change, vmPFC is robustly associated with behavior change, but several regions of the DMN are not).

Our analysis evaluated the flexibility of vmPFC to quantify how often it changed community affiliations over time. We tested whether individuals who demonstrated differential levels of flexibility in the vmPFC region showed corresponding variation in their intentions to quit smoking or smoking behavior in the month following the scanning session (for a total of 4 tests, including the Supporting Information using unsmoothed data, Cooper et al., 2018). vmPFC flexibility (displayed in Figure 5B) was not significantly related to changes in intentions using smoothed (continuous robust regression: *t*(38) = 1.55, *p* < 0.120) or unsmoothed data (Supporting Information). We next examined the relationship between vmPFC flexibility and behavior change (displayed in Figure 5C). vmPFC flexibility was significantly related to individual differences in smoking reductions 1 month after the scan, such that individuals with more flexible vmPFC network activity demonstrated larger reductions in their smoking behavior using smoothed (quartile robust regression, *t*(39) = −2.93, *p* < 0.005; continuous measure robust regression, *t*(39) = −2.85, *p* < 0.006) and unsmoothed data (Supporting Information); see Figure 5D and Supporting Information Figure S3. This suggests that the network interactions of vmPFC also capture an important component of its role in forecasting health outcomes.

## DISCUSSION

Previous research has identified the critical role of regions in several brain networks for persuasion and successful behavior change, but to date research has not examined whether interactions among these networks can account for individual differences in smoking outcomes. Interactions between pairs of regions have been related to message effectiveness and behavior change (Cooper et al., 2017, 2018; Dinh-Williams et al., 2014; Ramsay et al., 2013; Zelle et al., 2017), and we extend this work by utilizing a large-scale network approach. We employed recent advancements from network science to examine whether the synchrony within a network community (allegiance among the brain regions in the community) or between-network community interactions (flexibility of brain regions to coordinate across communities) accounted for lasting changes in smoking outcomes. We find that dynamics in two networks, the dafault mode (DMN) and fronto-parietal control (FPN) networks, may be relevant to smoking-related outcomes. We also find that more frequent network changes in a key node of the DMN consistently linked to predictions of behavior change, the ventromedial prefrontal cortex (vmPFC), are associated with reductions in smoking behavior.

### Relationship Between Network Allegiance and Changes in Smoking Intentions

Larger increases in intentions to quit smoking were related to reduced allegiance between nodes belonging to the atlas-defined DMN and FPN, particularly in analyses using smoothed data. In other words, there was lower consistent functional connectivity across the time course of the task within regions in each of these networks for those individuals who showed an increase in intentions to quit smoking. This reduction in network allegiance over the duration of the task may reflect differential recruitment of nodes in each of these networks to interactions with outside-network nodes, and it is plausible that this diversification of communication could support long-term intention change. A point of interest in future investigations will be identifying internetwork interactions that precede intention change, and examining whether these interactions involve entire functional network communities or subsets of these atlas-defined networks.

The finding of reduced allegiance within the atlas-defined FPN and DMN may be related to the possible division of these networks into smaller modules dependent on context and task demands. If these a priori networks are fractionated into modules that are more strongly connected to other networks than to each other, this could result in reduced intranetwork allegiance. Several studies identify meaningful subnetworks of both FPN and DMN; for example, Spreng et al. (2013) and Dixon et al. (2018) find separate types of nodes within the fronto-parietal control network, based on their interactions with other networks (Dixon et al., 2018; Spreng, Sepulcre, Turner, Stevens, & Schacter, 2013). The DMN has also been shown to be separable into subnetworks based on task-related functional connectivity (Dixon et al., 2017; Fornito, Harrison, Zalesky, & Simons, 2012), and both DMN flexibility (Stanley, Dagenbach, Lyday, Burdette, & Laurienti, 2014; Vatansever, Menon, Manktelow, Sahakian, & Stamatakis, 2015) and its connectivity with other networks (Finc et al., 2017) can change with task demands.

The FPN has been posited to change its connectivity patterns in response to changes in task demands to a greater extent than other functional networks (Cole et al., 2013), and such changes in FPN connectivity have been previously reported to correlate with greater changes in behavior. For example, reduced allegiance in hubs of the FPN predicted individual differences in learning (Bassett et al., 2015; Gerraty et al., 2018), as well as better performance on working memory and executive cognition tasks (Braun et al., 2015). Although these previous findings relating FPN connectivity changes to behavior come from other task domains, it is possible that the core process—learning—is similar to what participants are experiencing during exposure to persuasive messaging; in particular, the updating of beliefs during exposure to self-relevant information from the messages could be akin to learning. The results linking FPN to intention change, however, should be interpreted with caution, given that these results were less robust using unsmoothed data (see Supporting Information, Cooper et al., 2018).

### Relevance of vmPFC Flexibility for Smoking Behavior

We also find evidence for the importance of vmPFC flexibility. Individuals who displayed higher vmPFC flexibility, or switching of community affiliations, across the duration of the task also reported larger reductions in their daily smoking levels 1 month later. Activation in vmPFC during exposure to messaging has been repeatedly linked to long-term behavior change (Chua et al., 2011; Cooper et al., 2015; Falk et al., 2011, 2015; Riddle et al., 2016; Vezich et al., 2017; Wang et al., 2013), and it is possible that frequent community changes, corresponding to high flexibility, relate to the activation levels detected in prior work. The vmPFC has structural and functional connectivity with an array of regions in networks involving memory, affective regulation, and higher-order cognition (Amodio & Frith, 2006; Buckner et al., 2009; Price & Drevets, 2012; Roy, Shohamy, & Wager, 2012; Tomasi & Volkow, 2011). Our result suggests that the time-varying strength of these connections may influence long-term behavior. These results are also consistent with the possible broader relationship between reduced default mode allegiance and behavior change observed in our supplemental analyses (see Supporting Information, Cooper et al., 2018) using unsmoothed data; vmPFC is one key node in the default mode network, and greater flexibility in key nodes of the DMN would correspondingly be related to lower allegiance.

We also find that changes in smoking behavior and intentions are related to partially divergent metrics of neural dynamics. Several theories of health behavior posit that intentions to perform a behavior are an important precursor to behavior change, but that other factors also influence whether behavior change occurs (Ajzen, 1985, 1991; Armitage & Conner, 2001; Fishbein, 1979; Fishbein & Ajzen, 2011; Webb & Sheeran, 2006). This partial dissociation between intentions and behavior seems to be reflected in the brain in this study, where we find that some neural metrics related to changes in intentions are not related to changes in behavior, and vice versa. The present findings complement and extend previous neuroimaging studies of behavior change in which intention changes do not mediate the relationship between vmPFC activation and behavior change (Cooper et al., 2015; Falk et al., 2010, 2011), and also suggest that dynamics between subportions of the DMN may be worth exploring to bridge the neural underpinnings of intentions and behaviors. Together, these reports suggest that different neurocognitive processes during initial exposure may support the evaluation of intentions to perform a behavior, and the additional cognitions and actions that result in longitudinal behavior change. These results highlight promise in additional research to build a more a complete model of the relationship between immediate brain responses to persuasive messaging, and later outcomes such as self-reported intentions and behavior.

### Future Directions and Limitations

The underlying origin of individual differences in functional connectivity dynamics is an open question and an intriguing avenue for future research. Here, we expect that some individuals are more susceptible to persuasion through health messaging than others, and that we can detect this propensity by assessing network dynamics during the task. Furthermore, this could be due to differences in intrinsic dynamics of the networks of interest (i.e., a person-level factor), differences in the dynamics associated with processing the anti-smoking images (i.e., a message-level factor), or both (i.e., an interaction between the two). Although individual differences in networks may suggest that regional activity differences have a trait-like component, our inclination is to avoid a hard split between context dependence and traits, and instead consider the importance of varying timescales when considering context versus trait effects. That is, individual differences may appear trait-like when brain activity is observed in a single session or narrow time frame, but these between-subject differences may show more context-based effects when examined over longitudinal timescales and in response to different types of stimuli. We expect that network dynamics in the same individual might vary depending on the task presented (e.g., different message frames), although we do not directly investigate task conditions in this analysis; that is, we expect that network dynamics during this anti-smoking task may be related to changes in future smoking behavior, but not necessarily to other behavior domains, or even to different types of message approaches. Whether the effects we observe here are more strongly related to task-related processing or intrinsic dynamics could have differing implications for the design of more effective health messaging campaigns and broader questions about persuasion and influence, and hence provide valuable directions for future research; for example, the former would implicate the need for changes in the design of messages, and the latter might suggest participant-level interventions to improve receptivity to messaging (such as self-affirmation, as in Epton & Harris, 2008; Epton, Harris, Kane, van Koningsbruggen, & Sheeran, 2015; McQueen & Klein, 2006; Taber, Klein, Ferrer, Augustson, & Patrick, 2016). Thus, the extent to which network dynamics vary across different timescales and in response to different task domains is an important question for future work.

The difference in functional dynamics between task conditions, such as the negative and neutral anti-smoking messages presented here, might also provide further insight into the mechanism of the effects we identify in this report. Specifically, this could aid in understanding what message characteristics are important for changes in brain response and later behavior, and whether the effects we report in the current manuscript stem from stable trait-like neural tendencies, context-dependent shifts in brain dynamics, or an interaction between the two. However, constraints of the task design in this experiment (namely, short stimulus presentation times and a relatively small number of stimuli presented) prevented the estimation of functional connectivity dynamics separately between task conditions. Future work incorporating slower and longer task designs will provide insightful extensions of our results, advancing our understanding about message characteristics important for behavior change.

### Conclusions

Here we investigated the relationship between the time-varying nature of brain activity during exposure to anti-smoking messages and future changes in smoking behavior and intentions to quit. We found robust evidence that reduced allegiance within the atlas-defined DMN related to changes in intentions to quit smoking and that flexibility in the vmPFC related to changes in smoking behavior, and suggestive evidence that reduced allegiance in the FPN related to intention change. There is increasing recognition that consideration of brain networks and their dynamics, and not just activation in individual regions, is necessary for understanding human cognition and behavior; here, we show that metrics of functional dynamics can provide new information about individual differences in responsiveness to anti-smoking messaging. These results highlight the value in considering brain network dynamics for understanding message effectiveness and social processes more broadly.

## ACKNOWLEDGMENTS

We acknowledge Richard Gonzalez, Sonya Dal Cin, Victor Strecher, and Lawrence An for collaboration on a larger study relevant to this work; and Francis Tinney Jr., Kristin Shumaker, Li Chen, Nicolette Gregor, Becky Lau, Larissa S. Svintsitski, and Cole Schaffer for assistance with data collection.

## AUTHOR CONTRIBUTIONS

Nicole Cooper: Conceptualization; Data curation; Formal analysis; Funding acquisition; Methodology; Project administration; Software; Validation; Visualization; Writing – original draft; Writing – review & editing. Javier O. Garcia: Conceptualization; Formal analysis; Methodology; Resources; Software; Validation; Visualization; Writing – review & editing. Steven Tompson: Conceptualization; Data curation; Investigation; Software; Writing – review & editing. Matthew B. O’Donnell: Conceptualization; Data curation; Investigation; Resources; Software; Writing – review & editing. Emily B. Falk: Conceptualization; Funding acquisition; Project administration; Resources; Supervision; Writing – original draft; Writing – review & editing. Jean M. Vettel: Conceptualization; Funding acquisition; Resources; Supervision; Writing – original draft; Writing – review & editing.

## FUNDING INFORMATION

Emily B Falk and Jean M Vettel, Army Research Laboratory (http://dx.doi.org/10.13039/100006754), Award ID: W911NF-10-2-0022. PI Victor Strecher and Co-I Emily B Falk, National Institutes of Health (http://dx.doi.org/10.13039/100000002), Award ID: P50 CA101451. Emily B Falk, National Institutes of Health (http://dx.doi.org/10.13039/100000002), Award ID: 1DP2DA03515601. PI Emily B Falk and Co-I Nicole Cooper, National Institutes of Health/ National Cancer Institute and FDA Center for Tobacco Products (http://dx.doi.org/10.13039/100000002), Award ID: P50CA179546. The content is solely the responsibility of the authors and does not necessarily represent the official views of the NIH, the Food and Drug Administration (FDA), the Army Research Office, or the U.S. Government.

## TECHNICAL TERMS

Health behaviors: An individual’s actions and habits that relate to the maintenance and improvement of health and well-being or prevention of disease.
Persuasive messaging: In the context of public health campaigns, stimuli designed to motivate an audience to engage in health-promoting behaviors or refrain from unhealthy behaviors.
Large-scale brain networks: Neural systems that are widespread and distributed across the brain, and are commonly recruited as a coordinated unit during performance of a task or at rest.
Dynamic functional connectivity: Analytic measures that calculate synchronized patterns of activity between regional time series data.
Network communities: Clusters of brain regions that are identified as having similar functional connectivity patterns, defined using a data-driven technique.
Intentions: An individual’s readiness to perform a given behavior, assessed through self-reported surveys.
Behavior change: In public health, any effort (spontaneous or in response to an intervention or persuasive messaging) to change an individual’s actions to promote health and prevent disease.
Allegiance: A metric that quantifies the cohesion among regions in a network community across time.
Flexibility: A metric that quantifies how frequently a single region changes . its assignment across communities over time.
Nodes: The smallest unit of a network analysis, representing a cohesive brain region. Nodes can be defined based on brain anatomy or patterns of functional activation.
Multilayer community detection analysis: The process of assigning brain regions (or nodes) to network communities, when the network has “multilayer” observations, such as multiple measurements over time.
Brain network dynamics: Time-evolving interactions between brain regions thought to underlie cognition and action.
