# Supplementary material for: Time-evolving dynamics in brain networks forecast responses to health messaging
Source: Netw Neurosci. 2018 Nov 1;3(1):138–56. doi: 10.1162/netn_a_00058 (PMC6372021; doi:10.1162/netn_a_00058)
Supplement: Supplementary file 1 [file netn-03-138-s001.pdf]

Cooper, N., Garcia, J. O., Tompson, S., O'Donnell, M. B., Falk, E. B., & Vettel, J. M. (2018). Supporting Information for "Time-evolving dynamics in brain networks forecast responses to health messaging." *Network Neuroscience, 3*(1), 138–156. https://doi.org/10.1162/netn_a_00058

SUPPLEMENTAL MATERIALS

Supplemental analyses with unsmoothed fMRI data

Based on the sensitivity of network results to spatial smoothing, we repeated the analyses in the main manuscript using unsmoothed fMRI data, and find that the majority of results are robust to this change.

Allegiance in subnetworks relates to changes in intentions. Within the default mode network (DMN), allegiance was marginally related to intention change (continuous regression, $t= -1.89$, $p<.058$), such that greater intentions to change smoking behavior were related to lower DMN allegiance; using smoothed data, this relationship was statistically significant. DMN allegiance was also significantly related to behavior change (continuous regression, $t=2.36$, $p<.023$), such that larger reductions in daily smoking were related to lower DMN allegiance. Using smoothed data, this relationship was not significant.

Within the frontoparietal network (FPN), allegiance was not significantly related to intention change (continuous regression, $t=-1.32$, $p<.184$); this relationship was significant with smoothed data. Allegiance within the FPN was not significantly related to behavior change using unsmoothed data (continuous regression, $t=1.09$, $p<.290$) or smoothed data.

Results within the salience and subcortical networks are not substantively different using unsmoothed *vs* smoothed data. Using unsmoothed data, within the salience network, allegiance was not significantly related to intention change (continuous regression, $t=-0.59$, $p<.56$) or to behavior change (continuous regression, $t=1.19$, $p<.24$). Within the subcortical network,

allegiance was not significantly related to intention change (continuous regression, $t=-0.40$, $p<.68$) or to behavior change (continuous regression, $t=1.91$, $p<.07$).

VMPFC flexibility relates to later changes in behavior. Parallel to findings with smoothed data, flexibility of the VMPFC using unsmoothed data was significantly related to behavior change (continuous regression, $t= -2.61$, $p<.011$) and not significantly related to intention change (continuous regression, $t=1.56$, $p<.12$).

Supplemental figures

As presented in the main text using smoothed data, we find that reduced allegiance between nodes within the DMN predicted a greater increase in intentions to quit smoking. Figure S1 presents a scatterplot of the relationship between each individual's changes in intentions to quit smoking and allegiance in the DMN. DMN allegiance was adjusted for covariates in the continuous robust regression, namely Session 1 (baseline) intentions, personalization condition (Facebook vs Nimstim faces), gender, age, and ethnicity (white *versus* other).

Figure S1

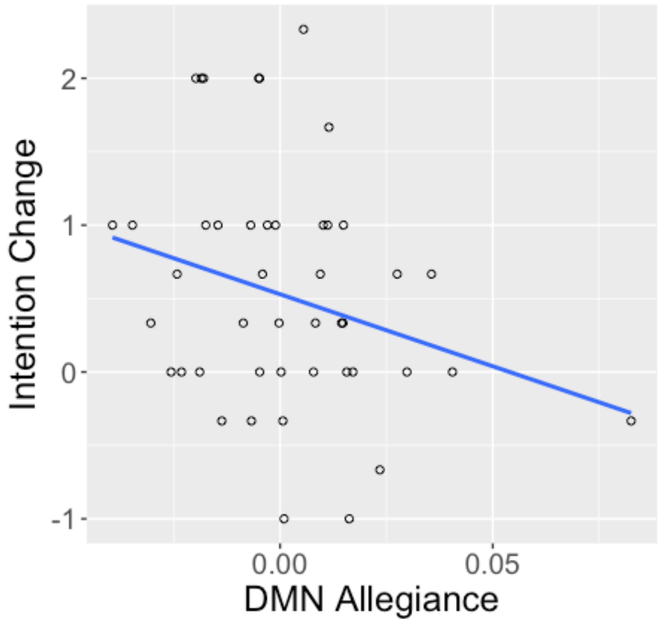

Reduced allegiance between nodes within the FPN also predicted a greater increase in intentions to quit smoking using smoothed data. Figure S2 presents a scatterplot of the relationship between each individual's changes in intentions to quit smoking and allegiance in the FPN. FPN allegiance was adjusted for covariates in the continuous robust regression, namely Session 1 (baseline) intentions, personalization condition (Facebook vs NimStim faces), gender, age, and ethnicity (white *versus* other).

Figure S2

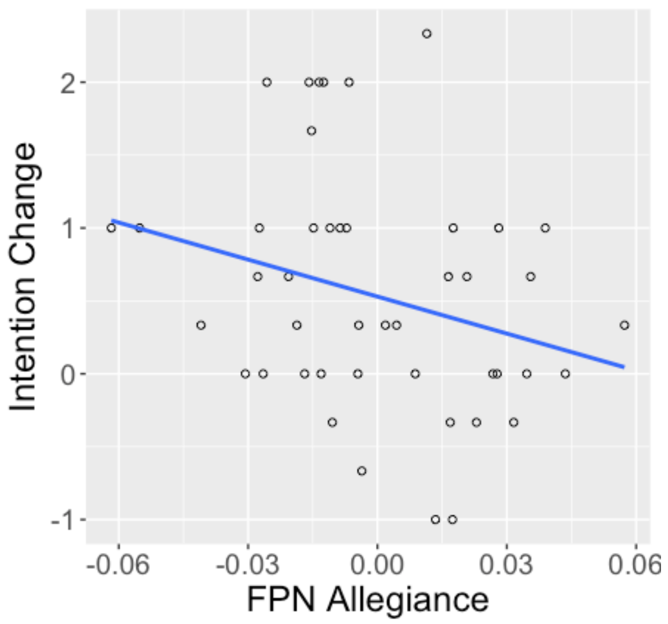

Finally, VMPFC flexibility was significantly related to individual differences in smoking reductions one month after the scan, such that individuals with more flexible VMPFC network activity demonstrated larger reductions in their smoking behavior. Figure S3 presents a scatterplot of the relationship between each individual's percent reduction in smoking and VMPFC flexibility using smoothed data. VMPFC flexibility has been adjusted for covariates in the continuous robust regression, namely personalization condition (Facebook vs NimStim faces), gender, age, and ethnicity (white *versus* other).

Figure S3

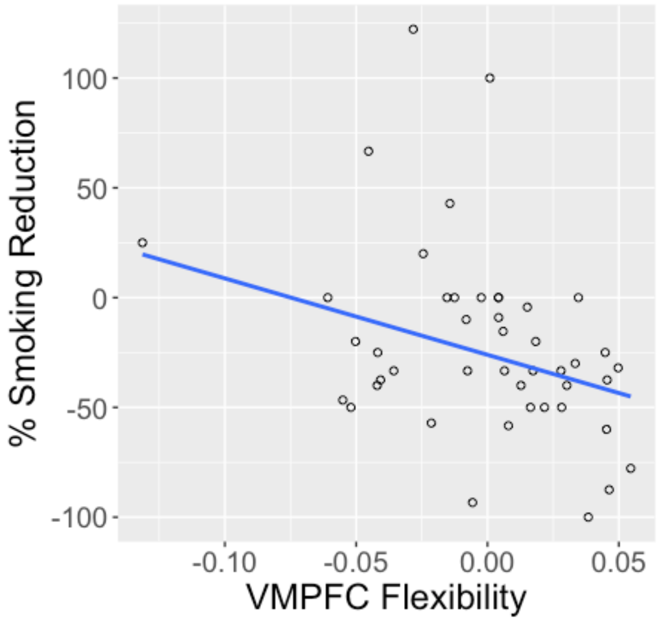

% Smoking Reduction
100
50
0
-50
-100
-0.10
-0.05
0.00
0.05
VMPFC Flexibility
